# Associating Vehicles Automation With Drivers Functional State Assessment Systems: A Challenge for Road Safety in the Future

**DOI:** 10.3389/fnhum.2019.00131

**Published:** 2019-04-24

**Authors:** Christian Collet, Oren Musicant

**Affiliations:** ^1^Inter-University Laboratory of Human Movement Biology (EA 7424), Univ Lyon, Université Claude Bernard Lyon 1, Villeurbanne, France; ^2^Department of Industrial Engineering and Management, Ariel University, Ariel, Israel

**Keywords:** driver functional state, automated vehicles, monitoring, drowsiness, level of automation, activation level, vigilance, road safety

## Abstract

In the near future, vehicles will gradually gain more autonomous functionalities. Drivers’ activity will be less about driving than about monitoring intelligent systems to which driving action will be delegated. Road safety, therefore, remains dependent on the human factor and we should identify the limits beyond which driver’s functional state (DFS) may no longer be able to ensure safety. Depending on the level of automation, estimating the DFS may have different targets, e.g., assessing driver’s situation awareness in lower levels of automation and his ability to respond to emerging hazard or assessing driver’s ability to monitor the vehicle performing operational tasks in higher levels of automation. Unfitted DFS (e.g., drowsiness) may impact the driver ability respond to taking over abilities. This paper reviews the most appropriate psychophysiological indices in naturalistic driving while considering the DFS through exogenous sensors, providing the more efficient trade-off between reliability and intrusiveness. The DFS also originates from kinematic data of the vehicle, thus providing information that indirectly relates to drivers behavior. The whole data should be synchronously processed, providing a diagnosis on the DFS, and bringing it to the attention of the decision maker in real time. Next, making the information available can be permanent or intermittent (or even undelivered), and may also depend on the automation level. Such interface can include recommendations for decision support or simply give neutral instruction. Mapping of relevant psychophysiological and behavioral indicators for DFS will enable practitioners and researchers provide reliable estimates, fitted to the level of automation.

## Introduction: the Promise of Automated Vehicles

Road traffic crashes represent a leading cause of death world-wide, more than 1.35 million lives each year, 48% of them in four-wheeled vehicles in Europe (World Health Organization, Global Status Report on Road Safety – Summary, 2018, pp. 2 and 6). Driving is a highly complex activity requiring considerable perceptual, physical, and cognitive demands on the driver (Sawyer et al., 2012) despite each of us has learned to drive a car. The human nervous system shows limitations in controlling much information in parallel and the human driver is one of main factors in over 90% of the crashes (Sabey and Staughton, 1975; Treat, 1977; Hendricks et al., 2001; Otte et al., 2009; Singh, 2015).

With the aim to increase safety, Advanced Driving Assistance Systems (ADAS) have progressively been integrated into vehicles and can either worn the driver or actively intervene in the vehicle operation. Many systems are now likely to assist the drivers both in usual driving (e.g., cruise control or electronic stability program) and in critical situations (e.g., antilock braking system, collision avoidance system). Merat and Lee (2012) considered that the automation process is now inevitable, and rapidly evolving vehicle automation will change vehicles more in the next 5 years than during the preceding fifty, until the driver may no longer be needed (Ivanco, 2017). To date, Waldrop (2015) underlined that automation is one of the main topics that could yield completely driverless cars within the next decade.

Until driverless cars are available, there is an urgent need to consider the effect of increasingly automated vehicles on the ability of drivers to operate the vehicle, monitor both environment and automation, and efficiently take over driving responsibility. These tasks require allocating mental resources to help the process of information from multiple cues (e.g., the environment, in-cabin signals). Ironically, while automation may free the driver from some of the traditional driving tasks, new operations are added (monitoring automation, responding to “take over” requests) and attention (the main focus of the driver mental resources) is expected to more frequently be directed to secondary tasks (Jamson et al., 2013; Llaneras et al., 2013). Thus, it is likely that automation will have mixed effects on the amount of mental resources drivers are now required to allocate. Hockey et al. (2003) refer to the general concept of “operator functional state” dealing with the operator ability to allocate the required resources to meet the task demands. The overall load originating from such demands impacts the operator functional state. Determining the extent by which the driver functional state (DFS) is suitable for the current driving challenge is most imperative.

The recording of physiological indices seems appropriate while considering the level of automation, but also environmental conditions (e.g., traffic density, type of roadways or weather conditions), driver characteristics (e.g., driving experience, automation intrusiveness, and trust in automation). All the aforementioned categories are likely to influence the DFS. The importance of selecting the appropriate physiological indices determines the reliability of assessing the DFS accurately. Future vehicles will need to incorporate a DFS estimation system that can potentially support interventions to maintain safety. Some examples for such interventions include switching to a more acceptable level of automation, issuing alerts to the driver or nearby road users, and applying interventions to increase arousal.

The main objective of this article is to review how associating vehicles automation with drivers functional state assessment systems. This literature review will be organized along with the five following research areas: We will first describe how different levels of vehicle automation should mediate the allocation of attentional resources to driving. The next section will detail the available methods of assessing the DFS. The complexity of assessing the DFS should point out the need to rely on different methodological solutions that must be integrated into a unique system. We will then propose a multimodal dataset acquisition requiring a close collaboration between the fields of engineering and behavioral neurophysiology thus leading to the redefinition of usual theoretical models. The whole of the preceding analysis will also have to take into account the singular characteristics of the drivers but also the external driving conditions. We will conclude by highlighting the contributions of our study to better understanding the relationships between vehicles automation and drivers functional state. We will also underline its limitations by acknowledging the path that remains to be done before we can propose complete autonomous driving solutions. This will not be done without the close collaboration between engineering sciences, neurophysiological and behavioral sciences.

### Levels of Automation and Allocation of Mental Resources

The Society of Automotive Engineers (SAEs) ranges vehicles automation capabilities from no automation (level 0) to complete automation (level 5). Level 0 accounts for most vehicles on the road today, where all driving tasks are manually handled. In level 1 (driving assistance), the vehicle has a single aspect of automation that assists the driver. Such automation level control either steering, speed (e.g., adaptive cruise control), or braking (e.g., automated emergency braking), but no more than one of these. In level 2 (partial automation), the vehicle can control both the steering and acceleration/deceleration, although the driver must always remain in complete control of the vehicle. This includes, among others, helping vehicles to stay in lanes and self-parking features. In level 3 (conditional automation) vehicles can make decisions for themselves such as overtaking slower moving vehicles. However, unlike the higher rated autonomous vehicles, this requires human override when the vehicle is unable to execute the task, or when the system fails. In this level, the driver must monitor automation and allocate attention to the driving as no information is provided about system failure. Level 4 (high automation) differs from level 3 in the sense that vehicles can intervene themselves in case of system failure. Thus, level 4 vehicles do not need human intervention in specific situations and will inform the driver on the need to take over in other situation as in occurrences of system breakdown or somehow underperformed or when in unfamiliar conditions (e.g., off-road driving, extreme weather). In level 5, complete automation does not require human interaction. Level 5 vehicles provide a much more responsive and refined service. These include off-road driving and other terrains that level 4 vehicles may not necessarily be able to detect or intelligently comprehend. In sum, the vehicle ability to monitor and “understand” the vehicle surroundings determines the level of automation. The main leaps in automation is between levels 2 and 3 in which the vehicle is already able to take complex tactical maneuvering decisions (e.g., changing lanes), and between levels 3 and 4 when human interaction is, in some circumstances, not required.

Whether one accepts the SAE scale of automation or proposes a different one, the discussion on the safety benefits of automation should consider the level of automation. While there is a broad agreement on the generally positive effect of automation, not all agree on the magnitude of this effect. As early as, Young and Stanton (2002) underlined that vehicle automation systems could reduce the required mental resources for driving and preserve safety by allowing the drivers to delegate some of their actions to the driving automation system. Therefore, drivers’ functions are shifting from operating their vehicles to supervising their automation (Shen and Neyens, 2017) and would require a lower level of general activation in the central nervous system and a more relaxed functional state. It is thus believed that monitoring a system cost less than operating it. However, no real comparison of the involvement of mental resources has been provided by the scientific literature and workload may be higher since the driver is now responsible for monitoring not only the environment but also the way in which the vehicle operates. Monitoring a highly complex system without a situated mental model or the requisite diagnostic skills may be proven challenging. Caldwell et al. (1994) defined ‘vigilance’ as the “*sustained readiness to detect and respond to changes in the environment*” (p. 14) and linked it to general arousal. On the one hand, arousal impacts vigilance in the sense that we cannot be vigilant if we are not sufficiently aroused. On the other hand, being activated does not imply that we adequately orient our attention toward useful indices, while inhibiting competing indices (distractors). People who actively generate responses in a system have greater situation awareness than those who passively monitor the same outputs performed by an automated agent (Metzger and Parasuraman, 2001). Many studies pointed out the risk for disengagement and distraction from the road scene and the driving task (Lewis et al., 2018). Increases in automation reduced driver vigilance as shown by braking reaction time, emergency steering (Saxby et al., 2013), and in decreased ability to maintain lane position (Shen and Neyens, 2017). Young and Stanton (2007) also observed decrements in attentional resources negatively affecting driving performance. Another aspect of impaired vigilance is the possible increasing involvement in secondary tasks (Shen and Neyens, 2017) that would possibly increase the whole allocation of mental resources but not due to the requirements of the main task.

**FIGURE 1:**
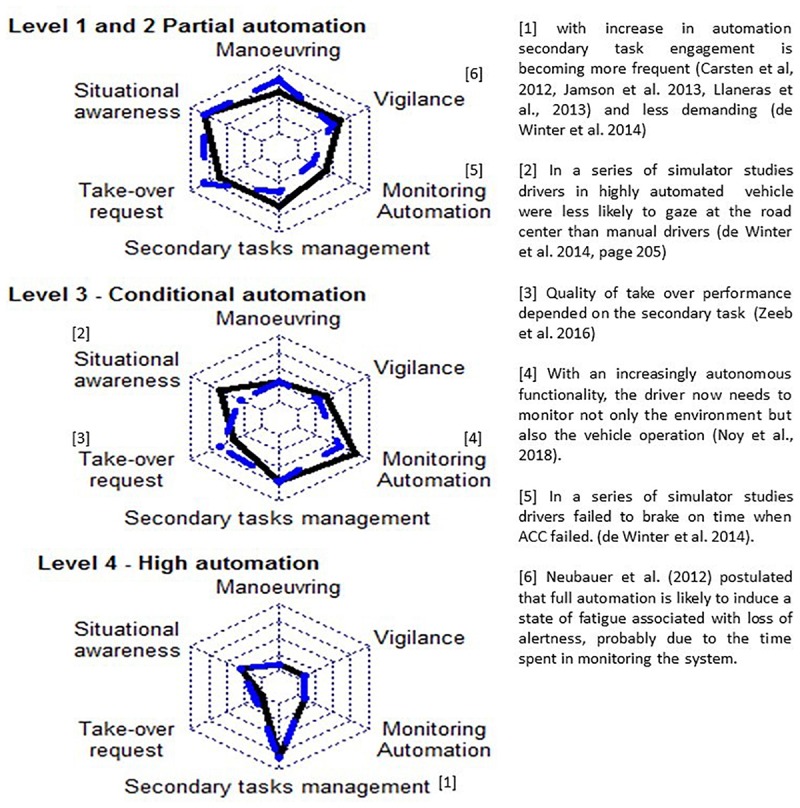
Illustration of required capacities (black) and available capacities (blue) by level of automation (subplot). Level 5 is not present in the figure since driver involvement is not required in complete automation.

The above review suggests that driver capacities as maneuvering, managing secondary tasks, situational awareness, vigilance in monitoring automation, and responding to take-over requests at least partly depend on the DFS. We argue that estimating the DFS (as we subsequently described in section “Estimating the DFS”) may have different strategies depending on the level of automation. To develop this argument, we refer to Figure 1 presenting three radar subplots, each corresponding to a different level of automation. Each radar subplot specifies a list of driving capacities (maneuvering, situational awareness…). Black line indicates the level of capacity that is required in each of the selected driving aspects. The Figure 1 presents how, with increased automation, maneuvering (i.e., correctly perform basic driving actions as braking and accelerating) and situational awareness capacities are becoming less and less required. The Figure 1 also presents the capacity of the driver according to his functional state (in blue).

If the DFS allows greater driving capacity (in blue) than what is required (in black), the probability of a crash remains low. However, a sudden increase in required capacity will also increase the risk of a critical situation. As the DFS can change from time to time, the reader should view the information suggested by the figure as an example for an arbitrary driver in an arbitrary time. To demonstrate that the figure presents plausible scenarios, we added references (indicated by the brackets []) for studies indicating when DFS (in blue) did not meet the requirements (in black). But clearly, more research is needed to accurately detect the relevant driving aspects, and their required capacities in the various automation levels. The information in Figure 1, therefore remains a schematic illustration of a possible future. Merat and Lee (2012) have also pointed out that little research has considered the consequences of high level of automation with most focusing on the effects of specific ADAS as lane-keeping or speed control (adaptive cruise control). This is an important concern despite some optimistic viewpoints (Merat and Lee, 2012; Waldrop, 2015), at this stage of autonomous vehicles development, automated driving is not yet reliable and safe (Dixit et al., 2016). Thus, research should study different levels of automation and accurately evaluate the effects of each on the DFS and consequently on drivers’ performance. For example, Eriksson and Stanton (2017b) tried to determine the time drivers needed to take-over control from a highly automated vehicle when confronted with non-critical driving scenarios.

**FIGURE 2:**
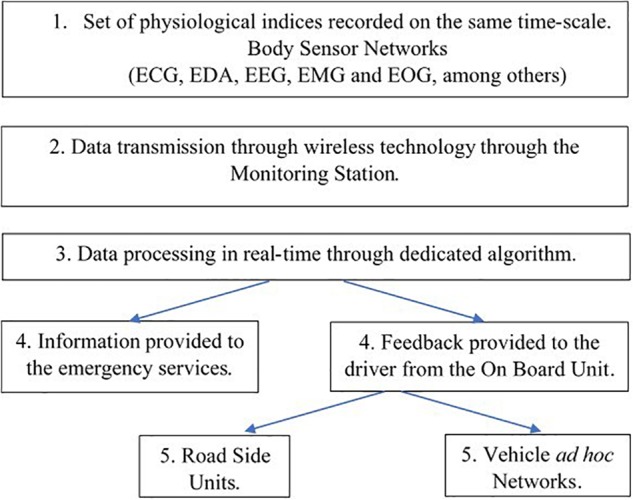
Steps from recording the driver’s functional state through body sensors until informing the emergency services, the driver himself and the environment. Adapted from Reyes-Muñoz et al. (2016), with permission of the editorial board of the journal “Sensors.” ECG, electrocardiography; EDA, electrodermal activity; EEG, electroencephalography; EMG, electromyography; EOG, electro-oculography.

As described in Figure 2, the ability to take-over is not required in automation levels 0 and 4 but may prove critical in levels 2 to 3. Whether the DFS is well-adapted when the need to take-over occurs is one of the key-points determining the “DFS/levels of automation” interrelationships. Several hypothesizes may be stated:

• The driver’s arousal level decreases under the automated mode as there is no need for him (her) to be aroused at that time. Taking-over may thus take more time than when the driver is less aroused and this is generally observed when taking-over occurs on urgent scenarios which could not be anticipated (Eriksson and Stanton, 2017a).
• Conversely, monitoring the automated system working may be demanding and may require an arousal level higher than at rest. In this case, the relationships between DFS and levels of automation are rather complex. When drivers were under reduced time constraint, Eriksson and Stanton (2017a) observed that taking over did not affect driving performance, however, with a large standard deviation thus attesting strong inter-subjects variations in behavior.

## Estimating the Dfs

Estimating the DFS can take several approaches: in low automation levels, the DFS is visible by monitoring kinematic indices of driving. Such indices are based on vehicle dynamic, e.g., the intensity of braking events, driving speed, lane position and distance to the lead vehicle. However, with increasing automation some of these actions are automated and may not reflect the DFS. Thus, the automated system operates well while the DFS is with low levels. Another, and perhaps more direct approach to estimate the DFS aspects is to tap into driver physiological indices as heart rate (HR), heart rate variability (HRV), skin conductance, and electroencephalography (EEG).

There is a large body of research that links driving performance with physiological arousal which clearly influence sensorimotor performance (Hockey et al., 2003). We cannot perform well without being aroused enough because the arousal level (tonic activity of brain structures associated to adequate muscles activation) determines the choice of useful information, its processing, and the motor response to be then implemented (Näätänen, 1973). Thus, functional state belongs to a conceptual framework including a quantitative dimension, i.e., energetic level supposing adequate (optimal) level of arousal which, in turn, influences a qualitative dimension, i.e., the ability to well process the information (adequate orientation of the attention, selection of useful cues, potential processing of concurrent information and inhibition of competing information). Boucsein and Backs (2009) elaborated an integrated model of arousal with four different levels, including sensory arousal, affective and memory arousal and arousal for action preparation. This is directly inspired from the earlier model by Näätänen (1973) supposing that performance directly depended upon both energetic and directional factors. On the basis of previous studies, general arousal is believed to impact behavioral efficiency since it involves the ability to mobilize the energy of the organism to face task requirement. Thus, DFS may be described through tonic variations of physiological indices, i.e., quantitative dimension associated with phasic physiological variations of the same indices, thus attesting information perception and processing (see Näätänen, 1973 for historical reference and Caldwell et al., 1994, for defining the activation/vigilance interrelationships).

In this context, we have the potential to assess the cost of taking-over from a highly automated vehicle (SAE level 3 and 4), the time needed for this and the quality of taking the vehicle back in hands (Payre et al., 2016; Eriksson and Stanton, 2017b). Carsten et al. (2012) studied to which extent driver attention to the road scene was affected by the level of automation provided to assist or to take over the basic task of vehicle control. Autonomous vehicles may thus be viewed with skepticism in their ability to improve safety when automated driving fails, or is limited, the autonomous mode disengages and the drivers are expected to resume manual driving (Dixit et al., 2016). An accurate and comprehensive approach to these factors is necessary to assess their effects on DFS. Thus, studying human-automated system interaction should consider the need to maintain attention during prolonged periods. In this context, the ability to detect and respond to rare and unpredictable events is of highly importance (roadway hazards that automation may be ill equipped to detect, according to Greenlee et al., 2018). Recording DFS at the same time would allow to verify whether it is adapted for safely driving (during both continuous monitoring and periods where taking-over is necessary). Finally, we should also include environmental factors in our analysis, e.g., the impact of traffic density and any additional task which could be performed simultaneously by the driver in highly automated driving (Zeeb et al., 2016). Here, we see that DFS determination depends on variable factors that are relatively difficult to identify. This tends to complicate the linking of the DFS with the level of automation of the vehicle.

In the following section, we will consider two main challenges:

(i) Which physiological indices are the best candidates to determine the DFS under naturalistic conditions?
(ii) How integrate redundant information into the recording system, redundancy ensuring its reliability?

A related requirement would be to eliminate false positives and negatives. If not, this will reduce driver’s trust in the system, or worse, drivers will consider the system unreliable. In this context, neuroergonomics^1^ can provide heuristic solutions since physiological indices can give useful information about DFS while being easily recordable with low intrusiveness. We could thus restrict the potential candidates to some central and peripheral indices (Lee et al., 2007; Clarion et al., 2009; Fernández et al., 2016).

### Physiological Indices From the Brain

At the central level, we should only consider ambulatory methods and not those from functional neuro-imagery (_f_MRI, MEG). Several tools with the ability to be used inside the vehicles are now available, e.g., electroencephalography (EEG – Lin et al., 2014; Damian et al., 2015) and functional near infra-red spectroscopy (_f_NIRS – Liu et al., 2016; Wang et al., 2016). EEG and _f_NIRS can provide information about DFS as they directly record intrinsic signals from the brain. Functional NIRS measures the cerebral microcirculation in the capillary networks and describes brain activations during actual driving sessions in real environments (Liu et al., 2016). Although it is premature to conclude that _f_NIRS will soon be integrated into real-time monitoring of DFS, several studies reported experimental designs both in simulated and actual driving (Liu et al., 2016; Wang et al., 2016).

Tonic variations of EEG waves are closely correlated to arousal states and can detect changes in brain activation. This is a real challenge to record EEG from inside vehicles (Caldero-Bardaji et al., 2016). Papadelis et al. (2007) requested sleep-deprived participants to drive in real field driving conditions and observed increase in brief paroxysmal bursts of alpha activity prior to severe driving errors. Anticipated EEG alpha bursts thus correlated with the risk to be involved in car crash. Damian et al. (2015) used mobile EEG to estimate the mental effort during a dual-task paradigm with EEG signal sent from wireless sensors during driving. Lin et al. (2014) assessed changes in drivers’ arousal, fatigue, and vigilance with reference to variations in task performance, by evaluating associated EEG changes. The same team (Lin et al., 2010) developed a brain-computer interface integrating a dual module for physiological-acquisition and signal processing. The embedded modules can monitor DFS in real time and provide biofeedback to the driver as early as the drowsy state occurs. Wireless sensors associated with real-time data acquisition/processing, and with a dedicated algorithm are the main tools of a system monitoring DFS. One remaining concern is related to sensors themselves as conventional physiological measurements techniques required to have the sensors in close contact with the human body. These could nevertheless interfere with driving operations as body segments can come into contact with some elements and as these are very sensitive to noise and artifacts (mainly caused by head movements). Sun and Yu (2014) described a non-intrusive driver assistance system which is likely to detect ECG or EEG signals through clothes or hair without direct skin-contact. Thus, the last feature of a brain–computer interface would be to remotely detect the physiological signals with no physical contact with human skin. The near future will probably see the development of such systems. We must acknowledge that asking drivers to affix sensors on their skin could be perceived as constraining, by the potential inconvenience to driving, by the time spent placing sensors, the latter may be made more difficult by the wearing of certain clothes. Considering that drivers would be required to wear a recording device on the head, which would be a prohibitive constraint for many people, data from EEG and _f_NIRS have low practical properties at that time. However, they can be supplemented by information from the peripheral nervous system, in particular the autonomic and motor nervous systems.

### Peripheral Physiological Related to Driving Performance

Several indices from the autonomic nervous system (ANS) are sensitive to time-dependent variations in arousal level and to external stimuli (Clarion et al., 2009; Brookhuis and de Waard, 2010; Johnson et al., 2011; Rigas et al., 2011). As the systems recording ANS activity are ambulatory and weakly intrusive, these are good candidate for DFS assessment (Rada et al., 1995; Axisa et al., 2004; Ramon et al., 2008). HR and electrodermal activity (EDA, skin conductance) increase with each incremental increase in cognitive demand (Mehler et al., 2012) and are closely related to functional state (Hugdahl, 1996). Among others, Porges (1995) and Hugdahl (1996) early promoted the role of the ANS in cognition. Porges (1995, for a review) underlined the role of the parasympathetic branch and particularly the vagus nerve on attentional processes. Several indices from the peripheral motor system respect the aforementioned criteria and may be pooled into three main categories (i) indices from electromyography (EMG) monitoring, with a special focus on muscles from the neck and the back of the driver, (ii) indices from the oculomotor system aimed at giving information on palpebral, dilation of pupils and eye-gaze related features, and (iii) indices from facial mimics through emotional face recognition.

Heart rate is a very easily recordable variable even without bodily placed sensors. Lee et al. (2007) elaborated a non-intrusive measurement of HR by integrating dry sensors into the steering wheel with a wireless design for data transmission (the safety belt can also provide a naturalistic way for recording HR). No differences from usual HR recordings were found with the design the authors conceived thus attesting its reliability. Beside the basal values, HRV has close links with fatigue and drowsiness detection (Li and Chung, 2013). Yu-Lung et al. (2016) recorded ECG from wireless thoracic sensors and process the cardiac signal using HRV. Several parameters (e.g., low-frequency power spectrum over high-frequency power spectrum or LF/HF ratio) were closely correlated to several changes in drivers’ behavior, particularly with the frequency of yawning episodes. By comparison with rest state or high level of arousal, HRV presents specific alterations during drowsiness episodes (Vicente et al., 2016). The authors claimed that incorporating drowsiness assessment on the basis on HRV signal may improve the existing car safety systems.

Electrodermal activity is closely related with arousal as it is directly under the control of the sympathetic endings innervating sweat glands without any influence of the parasympathetic branch, thus derogating from the well-described principle of double innervation (Collet et al., 2013). Importantly, EDA is a witness of sympathetic functioning alone. By confronted drivers to an unexpected critical crash avoidance situation, Collet et al. (2005) showed that EDA was a predictive index of drivers’ performance. The recording of EDA basal level along the whole session evidenced that drivers who avoided the obstacle pulled onto their traffic lane where those who exhibited the highest EDA basal values (about 30% above the reference EDA at rest). Conversely, the drivers who failed to avoid the obstacle showed a lower EDA level, at about 20% above the reference level at rest. Thus, drivers who performed well exhibited higher arousal and were more likely to perform adequately. More generally, when considering routine driving situations, there is a close positive relationships between EDA and cognitive demands (Mehler et al., 2012). Other indices can originate from basal EDA signal, e.g., the frequency of electrodermal responses was positively associated with decreased vigilance (Dementienko et al., 2001). When the drivers exhibited obvious signs of low vigilance, electrodermal response frequency decreased in parallel. We should nevertheless indicate that Dorrian et al. (2008) failed to evidence a relationship between EDA and participants state who were imposed one night of sustained wakefulness. While they rated increased levels of sleepiness and fatigue through paper and pencils tests, EDA did not present any difference between the reference period and the induced sleepiness and fatigue state. EDA usually range from 1.5 to 70 μSiemens and data processing should be done with caution due to the high differences among people. Preventing metrologic errors due to individual differences may easily be overcome by normalizing data. Another way to increase reliability is to simultaneously record other physiological indices. This is usually done when experiments are designed to study complex human brain functions, such as DFS. There are thus many contributions presenting a data set of physiological indices (Rada et al., 1995; Ramon et al., 2008; Clarion et al., 2009; Lanatà et al., 2015; Taamneh et al., 2017). Lanatà et al. (2015) evaluated DFS by analyzing ANS changes through HR, EDA, and respiratory frequency along with performance indices of steering wheel angle corrections and response time. This study was performed under simulated driving conditions, but Healey and Picard (2005) already provided evidence of physiological recordings under actual driving conditions. They reported that EDA and HR were the most closely correlated with driver strain. Physiological monitoring could thus provide a continuous assessment of how different driving contexts but also driver emotional states affect DFS. These studies clearly show the ability of peripheral physiological variables to closely correlate with the DFS. They can be supplemented by behavioral variables.

### Behavioral Indices Related to Driving Performance

Reyes-Muñoz et al. (2016) identified five behavioral indices that are close correlated to drowsiness, i.e., frequent yawning, frequent eye-blinking, pupil movement (gaze), head movement and facial expression. Fernández et al. (2016) also provided a thorough review focused on the role of computer vision technology applied to the development of monitoring systems. They considered that seven factors could evaluate the DFS with highly acceptance: reliability, real-time performance, low cost, small size, low power consumption, flexibility, and short time-to-market.

Yu-Lung et al. (2016) elaborated an intelligent driver assistance system including a camera in front of the driver for facial monitoring. Frequency of yawning was one of the main index predicting the occurrence of drowsiness (see also Sigari et al., 2014). Fernández et al. (2016) considered that the eyes are the most remarkable information sources in face analysis as they reflect affective states and focus of attention. There are nevertheless several methodological obstacles to overcome before providing a reliable set of information from the visual system (e.g., keeping the camera closely orienting on the eyes despite head movements). Song et al. (2013) described the main factors challenging accurate eyes localization, due to variations in facial expressions, variations of gaze direction, head/eyes movement coordination and surrounding lighting. The measures may be hindered by the wearing of glasses especially sunglasses and makeup (Fernández et al., 2016). Eye-blink and eyelid closure are of interest in detecting early signs of drowsiness, as these may be captured by a set of cameras placed on the dashboard (Hu and Zheng, 2009) and blinking has been reported to change during cognitive distraction phases (Fernández et al., 2016). Data acquisition and processing are provided by the seeing machines which *“continuously measure operator eye and eyelid behavior to determine the onset of fatigue and micro sleeps and deliver real-time detection and alerts”* (Fernández et al., 2016, p. 25 of 44).

Recordings of EMG activity have a high potential to bring information about the DFS. The alteration of muscles function may be associated with impairment in driving abilities and fatigue. Surprisingly, there are little scientific contributions from this field. Fu and Wang (2014) showed that the peak factor and the maximum of the cross-relation curve, two indices from surface EMG of the biceps femoris, were related to drivers’ fatigue. EMG recorded from the neck and the back muscles are likely to provide information about sleepiness and driver fatigue. However, muscles activity is difficult to capture given the driver’s sitting position, with the risk of sensors contact with the seat or headrest, thus affecting data reliability. Finally, head movements recordings by embedded cameras can provide similar information to that provided by EMG. Methodological difficulties may explain the weak number of works involving EMG in actual driving. Despite behavioral indices of drowsiness occurrence are promising methods, Sahayadhas et al. (2012) underlined that the reliability and accuracy of driver drowsiness detection by a set of physiological indices is higher than that coming from other methods such as vehicle-based measures and behavioral measures.

## An Obvious Requirement: a Multimodal Dataset Acquisition

Beside the methods used in laboratories, the challenge is to propose pragmatic, integrated systems, including a set of behavioral and physiological indices, simultaneously recorded in real time, both from the driver and the environment. This involves selecting indicators for their reliability and complementarity. Maglione et al. (2014) simultaneously recorded high resolution EEG data associated with heart and eye blinks rates. Then, fusion of data provided a robust method in studying complex human activities, involving several functions (Noori and Mikaeili, 2016). Rigas et al. (2011, 2012) described a set of physiological signals (ECG, EDA, and respiration) associated with driving history from the GPS and the vehicle’s controller area network-bus (CAN) data. They incorporated these data into a Bayesian network (BN) and estimated that the system could detect stressful events with an accuracy of 82%. The development of an intelligent algorithm capable of recognizing the drivers’ affective state was proposed by Singh et al. (2013). It was based on several physiological indices including EDA and blood flow through photo-plethysmography during on-road driving. Their neural networks are believed to predict DFS with a nearly 90% average precision. According to Reyes-Muñoz et al. (2016), recording physiological variables for DFS assessment could allow rescuers to make a faster and more accurate diagnosis in case of an accident, if the data is transmitted to the rescue services (Figure 2 summarizes the successive steps from data acquisition/processing until provided feedback to the driver and eventually to the road control or emergency services).

## Drivers’ Individual Features and External Conditions

In addition to the variables used to evaluate the DFS, we must take into account two intrinsic factors, the individual characteristics of the drivers and the external driving conditions. One of the main concerns in providing feedback to the drivers is their high behavioral variability. There is thus considerable dispersion around the median behavior depending upon driver’s characteristics in age, gender, driving experience and perhaps more importantly their psychological particularities or specific individual traits. Ranney (1994) underlined that the inherent variability of human behavior may be responsible of errors associated with an important rate of roadway crash causation. By comparison, systematic errors attributable to the well-known limits of the human information-processing system seems rarer. In fact, all driving activities are believed to associate fast sensorimotor and automatic components with slower and more deliberate controlled cognitive processes. This refers to intra-individual behavioral variations as a function of time (according to specific individual differences, mental state and environmental context). Determining the boundaries around which the automated system could provide useful information to the drivers, i.e., with a high probability to take it into account for its meaning, is a key-component to be resolved in the next future. Little is known about how personality traits lead people to consider or ignore a given information. If the personality traits are stable features, and can be taken into account, the emotional states are more transient and therefore more difficult to detect. Yet, we know that they influence driving (Chan and Singhal, 2013) with a high probability of diverting the driver from the road scene. Another important concern is about how old drivers perceived the integration of more and more automated devices into their vehicles. First, Molnar and Eby (2017) question their motivation for technology use and assigned meanings. Second, they wonder whether the in-vehicle monitoring technology will be used and how transfer of control between automated and manual driving would occur in the elderly population. The role of trust in automation and its interaction with practice of partially or fully automated vehicles is also a key-variable. Payre et al. (2016) observed that drivers who had high trust in the automated vehicle exhibited longer reaction time when they were required to take-over by manual control recovery. Thus, over-trust may have deleterious effect on performance, a well-known effect of what high-technology is believed to bring (Collet et al., 2005). Overconfidence in vehicle equipment made drivers less efficient and this correlated well with a weak arousal level. This is well summarized by Endsley (2017): *“more autonomy is added to a system and its reliability and robustness increase, the lower the situation awareness of the driver and the less likely that he will be able to take over manual control if needed.”*

**FIGURE 3:**
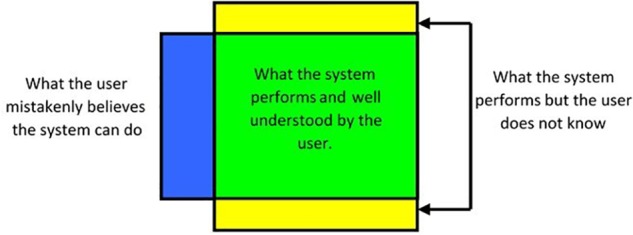
Real assistance capacity of an automated system based on the knowledge of the user.

These examples clearly advocate for education in the use of automated systems. Strauch (2017) deplores that drivers have not gained enough expertise needed to effectively operate automated systems. Instead, they are forced to obtain the expertise *ad hoc* during system operations. We nevertheless suppose that the in-vehicle intelligent devices should identify the driver (through face identification), retrieve his previously stored profile from its data to then intelligently prescribe specific accident prevention tools and driving environment customizations, as proposed by Sawyer et al. (2012). At least, we should be informed and trained about how the automated device works so that we can improve take over whenever necessary. We should also change our representation about automated systems, as suggested by Figure 3 where the interactions with them can include three modes:

• The first is the adequacy when the driver understands some features of the system and uses them (in green).
• The second corresponds to the disjunction between the partial knowledge of system functionalities by the driver and the representation that s/he has some. In fact, the user does not know some features of the automated system and thus can obviously not use them (in yellow).
• The third is false representations as the user wrongly thinks that the automated system can fill certain functions while it cannot (in blue).

Reducing the discrepancy between drivers’ representation of the system functioning and its actual abilities and functionalities (e.g., levels of automation) would probably imply to redefine the procedures of learning to drive.

## Conceptual Changes for Current Models of Driving Performance and Learning

Over the years, human factors research proposed several models for driver performance (Shinar, 1978; Michon, 1985; Endsley, 1995; Fuller, 2005; Wickens et al., 2015). These regard the driver as an information processing unit. Such information and attention models describe how the driver obtains data using his/her sensorial systems (vision, audition, etc.), process them to gain significant insights, apply a decision-making mechanism (e.g., slow down), adapt the decision to the actual context (e.g., adjust the braking intensity) and execute the decision with success determined by his/her abilities. According to these models, the driver limited capacity to collect and process the information from the environment explains driver error and misjudgment.

**FIGURE 4:**
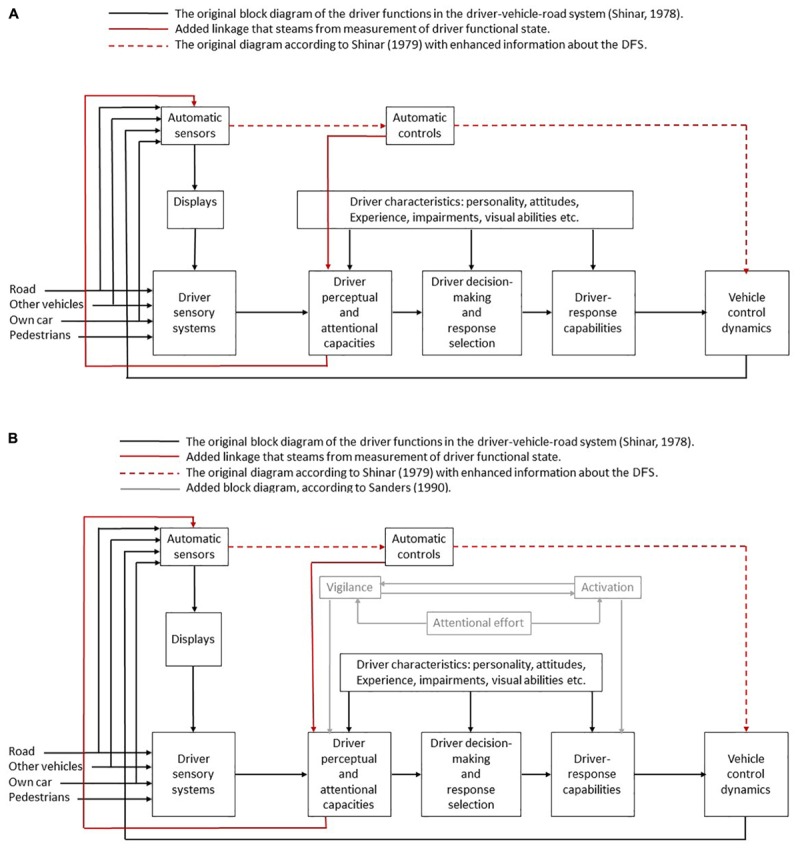
**(A)** A limited-capacity model of driver information processing (adapted from Shinar, 1978) with added paths for DFS. Reproduction with permission of the author. **(B)** A hybrid limited-capacity model of driver information processing paths for DFS (adapted from Shinar, 1978 and Sanders et al., 1990). Here, we integrate all the factors that are supposed to make the DFS varying. The operations of information processing (perception, attention, memory access, decision making, and motor adjustments) require the mobilization of mental resources (attentional effort) and, thus change the DFS so that the driver is able to drive efficiently and safely. The DFS can be evaluated by a set of physiological and behavioral indices, and adjust as a function of the level of vehicle automation.

Here, we examine a model that was developed almost 40 years ago by Shinar (1978). We show how, in some ways, this model is still useful, and how it should be updated to incorporate new abilities to monitor the DFS. We explain how such updates may have potential safety benefits. In Figure 4A, the black lines depict the original connections in the model by Shinar (1978). The red dashed lines depict original connections that now serve to transfer information and driving decisions about the DFS as well as information and driving decisions that stem from knowledge about the environment. The red lines did not appear in the original model and represent new contributions. The original model (black lines) describes how the driver sensory system receives various cues, the information is then processed according to the driver perceptual and attentional capabilities to facilitate decision making and response. These cues do not include DFS information. Next, the driver response impacts the vehicle dynamics. It is interesting that so long-ago, Shinar (1978) included a path for an autonomous system that can (1) display feedback to the driver (e.g., as done by level 0 systems as collision warning systems and navigation systems), and (2) control vehicle dynamics (e.g., as done by level 1 systems as adaptive cruise control and automatic emergency braking system). Despite the time passed since this model formulation, similar modes still guide research teams in international meetings (Keith et al., 2005). The red lines represent the transfer of physiological cues related to the DFS. Determining the DFS can be based on ECG (HRV), EDA, and EEG. The driver himself may be aware of his physical and mental states through sensory feedbacks (e.g., fatigue, or other temporal impairments), and can decide to take measures to adjust (e.g., stop for a rest) or, in the near future, to engage an automated driving mode.

However, with the advances of physiological monitoring technology, these data can be picked up by electronic sensors (e.g., sit sensors or wearables). In automation level 0, the system can display this information back to the driver (e.g., a coffee icon can suggest that s/he should rest, other displays can warn about increase in mental effort, decrease in general activation or any other physiological condition). In levels 1–3, an automatic action can take two directions: the first is to increase driver perceptual and attentional capabilities. Numerous interventions can be suggested here. When mental workload is high, such intervention can lower the volume of the music, adjust air condition, shut off infotainment and message. In very low mental workload, the automated system can propose Trivia Games and even increase the volume of the music. On the second path, the autonomous system can control the vehicle dynamics to reduce demands from the environment. An interesting study by Hajek et al. (2013) investigated the safety benefit and acceptance of an adaptive cruise control that selected the optimal safe distance to the lead vehicle according to the DFS (estimated by the driver physiological indices). Another example is the ability to use physiological indices to predict intentions for emergency braking (Kim et al., 2015), such an ability may be use either to trigger the automatic emergency braking system (level 1) or to release a distress signal to the autonomous system (level 5) which can learn to avoid such stressful conditions for its driver (supervisor) in the future. In sections two and four, we mapped several physiological and behavioral indices that can be used to estimate these closely related aspects of driving. In Figure 4B, we offer that physiological indices for activation and vigilance also have a link for the automated sensors.

## Conclusion

Research into the effects of automation on DFS is expending due to understanding that in the near future, the human factor will remain an important component in driving and in monitoring automation. This manuscript points on: (1) The need of accurately assessing DFS, (2) Estimating the DFS may have different strategies given the level of automation, (3) Estimating the DFS can infer on interventions that are also related to automation (e.g., switching between levels of automation). Based on these understandings (points 1–3), we reviewed methods for estimating the DFS and described the potential characteristics of an in-vehicle system. With regard to the first point, commuting executive functions usually performed by the driver to ADAS is likely to make her/him less concentrated on driving. The driver can monitor the system working or be engaged in other tasks with a connection with driving (supervising the route plans through the GPS) or not (reading or phoning or discussing with other passengers). Depending on these different activities, DFS may stay at a level comparable to that required for driving (parallel activity with the same demand as driving) or can change drastically and reach a level incompatible with driving (decrease in arousal level). This seems of particular importance in case of sudden need of taking-over. With regard to point 2, the extent by which driver’s DFS remains at an adequate level is still pending and depends on different but interrelated factors (level of automation, driving conditions, driver’s personality). With reference to point 3, an acceptable alternative would be to propose an intelligent system where we would choose the level of automation according to the objective of our trip (professional or leisure trip) of our state of fatigue (strong delegation or conservation of driving control) or conditions of external driving (traffic density, weather conditions). For example, traffic density has been shown as influencing the way in which the take-over is performed, with higher time to proceed and less accuracy when traffic is dense (Gold et al., 2016).

An integrated system capable of monitoring DFS in real time, should be based on several physiological indices recorded inside the vehicle. This would probably be the best way to ensure safety provided that it is built on sufficiently powerful algorithms capable of including all the driving scenarios that can potentially occur, thus depending on the external conditions (where driving takes place with traffic and weather conditions). It should also be able to provide useful feedback, from simple information about his functional state to even delivering graduated alerts, depending on their severity and urgency. Finally, we contributed to show how monitoring DFS can serve to update existing driving performance models to provide feedback to drivers and to automatically adjust autonomous behavior.

## Author Contributions

OM and CC conceptualized and designed the study, organized the manuscript, and wrote the first draft. They contributed equally in writing the manuscript.

## Conflict of Interest Statement

The authors declare that the research was conducted in the absence of any commercial or financial relationships that could be construed as a potential conflict of interest. The reviewer JV and handling Editor declared their shared affiliation.

## Footnotes

1 Mehta and Parasuraman (2013) defined neuroergonomics as an emerging science studying human brain indices in relation to performance in a workplace and everyday settings.
